# Polypropylene Dissolution Kinetics: Effects of Solvent, Temperature, and Particle Size

**DOI:** 10.3390/polym17233213

**Published:** 2025-12-02

**Authors:** Paschalis Alexandridis, Ali Ghasemi, Marina Tsianou

**Affiliations:** Department of Chemical and Biological Engineering, University at Buffalo, The State University of New York (SUNY), Buffalo, NY 14260-4200, USA; alighase@buffalo.edu (A.G.); mtsianou@buffalo.edu (M.T.)

**Keywords:** polyolefin, polypropylene, diffusion, polymer dissolution, crystallization, plastic recycling, chemical recycling

## Abstract

Polypropylene (PP) is widely used and currently very little recycled. A promising method for recycling the PP present in plastic waste involves its selective dissolution and subsequent separation from undissolved compounds. We address here the fundamentals of PP dissolution. Specifically, we present a model that describes the different phenomena involved in the dissolution of semicrystalline PP and validate the model with the experimental results on the decrystallization and dissolution kinetics of PP pellets. The model provides detailed time-resolved and position-resolved information on composition (i.e., crystalline PP, amorphous PP, and solvent) and solvent diffusivity (which depends on composition) across the dissolving polymer particle, in different solvents and temperatures. Such information is unavailable experimentally or difficult to obtain. The key fitted parameters that capture decrystallization and polymer chain disentanglement decrease with increasing temperature following an Arrhenius relationship, with activation energies higher than that for crystallization and comparable to that for melt viscosity. Both decrystallization and dissolution times increase with particle size. For smaller particles, decrystallization and dissolution occur nearly simultaneously, while for larger particles, their interior remains solvent-poor and crystalline for longer times. This work offers insights into the interplay of decrystallization and polymer chain disentanglement during the time-course of PP dissolution. Further, this work facilitates the design and optimization of a dissolution–precipitation recycling process that can unlock value from the million tons of PP annually that is currently being landfilled or incinerated following its use.

## 1. Introduction

Polyolefins are the most widely used polymers, with annual production exceeding ~200 million metric tons [1], due to their desirable properties, such as durability, barrier, optical, and processibility, their cost affordability, and their ability to change their intrinsic properties when formulated with additives [2,3]. Polyolefins are most commonly used as packaging materials [4]. In particular, polypropylene (PP) is used to make food packaging, sweet and snack wrappers, hinged caps, and microwave containers and also other products such as pipes, automotive parts, appliances, and syringes [5]. PP may attain isotactic, syndiotactic, and atactic configurations; isotactic PP (iPP) and syndiotactic PP (sPP) are semicrystalline, whereas atactic PP (aPP) is essentially amorphous due to the irregular arrangement of its side groups [6]. Most packaging is single-use, which means that the majority of the 79 million metric tons per year [7] of PP becomes part of plastic waste. Just 3% of PP is recycled [8]. Besides the loss of resources associated with the PP material that this entails, PP that ends up in landfill occupies land that could find better uses, while PP that is mismanaged and leaks into the environment contributes to pollution, including the generation of microplastics [8,9,10]

Polypropylene is challenging to recycle through mechanical recycling [11,12]. It degrades under heat, losing strength and flexibility during extrusion, resulting in reduced molecular weight, lower melt-flow rate, and compromised thermal stability compared to virgin PP. Recycled PP often fails to meet food-grade standards without costly additives; hence its recycling rate remains low [7,8]. These limitations underscore the need for alternative approaches. Advanced or chemical recycling can recover value from PP waste streams that are contaminated or degraded, but such recycling typically involves pyrolysis, which breaks down PP into short hydrocarbons [13,14]. To retain its molecular structure, PP can be recycled by dissolution–precipitation. In this method, plastic waste is first dissolved in a selective solvent that dissolves the target polymer, then the immiscible solids are filtered out, and then the target polymer is precipitated from the solution by cooling down, adding anti-solvent, or evaporating the solvent. In comparison to other chemical recycling methods, dissolution–precipitation is true recycling as it recovers polymers and requires much less energy and emits less greenhouse gases than pyrolysis. Moreover, dissolution–precipitation can remove a broad spectrum of additives [15,16,17].

The potential of dissolution–precipitation for recycling polypropylene is demonstrated by its adoption in industrial practice. For example, APK’s Newcycling process in Merseburg, Germany, operates a 20,000-ton-per-year facility that selectively dissolves plastics to recover virgin-quality PE and PP from mixed waste streams. As of October 2024, APK is a wholly owned subsidiary of LyondellBasell (Rotterdam, The Netherlands), which is leveraging this technology to produce high-purity recyclates [18,19]. PureCycle Technologies (Orlando, FL, USA) uses dissolution–precipitation to remove color, odor, and contaminants and produce Ultra-Pure Recycled (UPR) resin with properties comparable to virgin PP. The PureCycle facility in Ironton, Ohio, is projected to reach an annual capacity of ~48,600 tons of recycled PP resin [20], and a 59,000-ton-per-year facility in Antwerp, Belgium, is planned [21].

Despite PP being one of the most widely produced polymers and the feedstock of industrial-scale dissolution–precipitation recycling plants, surprisingly little is known about its dissolution. Back in the 1970s, Blackadder & Le Poidevin published a four-part series that remains the most detailed account of PP dissolution [22,23,24,25]. They described the dissolution process as occurring in stages: initial solvent diffusion and swelling, followed by the disruption of crystallites and chain disentanglement. They further noted that, depending on polymer density and stirring speed, the rate-controlling step “can be imagined” as either solvent diffusion and crystallite destruction or the disentanglement of the amorphous phase. In addition, they reported that the dissolution rate increases with decreasing PP molecular weight and that solvent effectiveness scales with molecular size, viscosity, and thermodynamic compatibility. Yet, these studies were limited: they provided no explanation of why or how the rate-controlling step shifts under different conditions such as temperature or solvent type, and they only provided an empirical discussion of solvent effects. Particle size was not considered nor was the evolving internal structure of dissolving PP. Remarkably, nothing was published in the decades that followed until, very recently, Ferchichi et al. revisited PP dissolution using in situ Raman, Attenuated Total Reflectance–Infrared Spectroscopy (ATR-IR) and Differential Scanning Calorimetry (DSC) [26,27]. This work offered valuable experimental observations of solvent-induced decrystallization but remained descriptive, without addressing the decrystallization of PP during dissolution; without an analysis of particle size, solvent, or temperature effects on the rate-controlling steps; and without connecting observations to predictive kinetics.

It becomes clear from the above that, despite the fundamental importance and practical relevance of PP dissolution, a lot remains to be learned. The following questions are of particular interest: What are the molecular-level mechanisms that are involved in the dissolution of PP? What is the influence of solvent type and temperature on the dissolution mechanism and kinetics? How can we incorporate the above into a quantitative model that can be used to make predictions and evaluate “what-if” scenarios regarding PP dissolution mechanisms and kinetics?

To address these open questions, here we generate experimental results on PP decrystallization in decane and decaline across a range of temperatures to complement data on the amount of PP dissolved from the literature; develop a model to describe the dissolution of semicrystalline polymer spherical particles; validate the model against the experimental results on PP decrystallization and dissolution; probe the effects of temperature and solvent type on PP decrystallization and disentanglement on the basis of fitted parameters and model predictions; and examine particle size effects on decrystallization and dissolution.

The novelty of this work includes experimental data on the decrystallization time of PP in two solvents and a validated model that quantitatively describes the physical processes taking place during the dissolution of semicrystalline PP and can predict macroscopic observables (e.g., time for complete dissolution, particle size evolution) and reveal microscopic information (e.g., spatial and temporal compositions and solvent mobility within the dissolving particle) for PP dissolution across a range of particle sizes, solvents, and temperatures. In addition to its fundamental contributions, this work informs the design of dissolution–precipitation processes for recycling PP.

## 2. Modeling

The dissolution of semicrystalline polymers involves solvent diffusion in the polymer, disruption of crystalline domains, swelling of amorphous domains, disentanglement of polymer chains from the solid matrix, and diffusion of disentangled chains in the solvent [28,29]. Significant gaps remain in quantitatively capturing the influence of polymer type, solvent type, and temperature on the key steps of dissolution such as polymer and solvent diffusion, decrystallization, and polymer chain disentanglement. Therefore, these key processes merit systematic investigation.

A model that can describe the dissolution behavior of semicrystalline PP pellets is presented here, building upon our group’s previous work on the dissolution of cellulose fibers [30]. The PP pellets are considered spheres, which simplifies geometry while preserving volume accuracy [31,32]. The model incorporates free volume theory to quantify temperature- and composition-dependent variations in solvent diffusivity within PP pellets. The robust predictive capability of this model is validated against experimental data on the dissolved fraction and the time required for the full decrystallization of PP pellets under various temperatures and solvent types, according to the Section 4. This comprehensive approach not only investigates the fundamental dissolution mechanisms of PP pellets but also offers practical guidance for optimizing dissolution parameters relevant to large-scale plastic recycling and processing applications.

**Figure 1:**
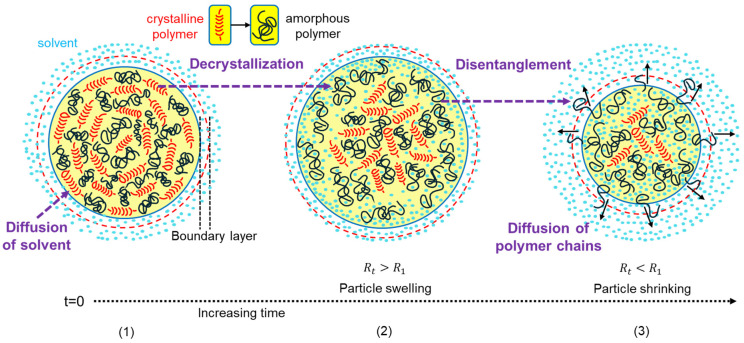
Schematic of dissolution process of spherical semicrystalline polypropylene pellet in solvent: (**1**) Initial state with homogeneous distribution of crystalline lamellae and amorphous chains within particle. (**2**) Solvent diffusion into amorphous polymer regions, causing polymer swelling and decrystallization of crystalline domains. Solvent concentration gradient is established, leading to particle surface decrystallization while core remains crystalline-rich. (**3**) Disentanglement of polymer chains and their diffusion into solvent (indicated by the black arrows), resulting in gradual particle size reduction as dissolution progresses.

Figure 1 schematically presents the progressive dissolution of spherical semicrystalline PP particles: starting from solvent diffusion into the polymer matrix, through swelling and crystalline domain disruption, followed by chain disentanglement and the diffusion of disentangled chains into a gel-like boundary layer, and ending with the gradual shrinkage of particles as dissolution proceeds. In what follows, the governing equations and underlying assumptions for modeling the dissolution kinetics of spherical PP particles are presented.

### 2.1. Solvent Diffusion in Polymer

Solvent diffusion in spherical semicrystalline polymer particles follows radial diffusion that can be modeled by Fick’s second law in spherical coordinates, accounting for solvent concentration gradients as a function of radial distance (*r*) and time (*t*):

(1) $$\frac{\partial \emptyset_{s}}{\partial t}=\frac{1}{r^{2}}\frac{\partial }{\partial r}\left({r^{2}D}_{s}\frac{\partial \emptyset_{s}}{\partial r}\right)$$

∅*_s_* is the volume fraction of the solvent in the polymer. *D_s_* is the solvent diffusivity in the polymer, considered here to vary with temperature, the specific solvent–polymer combination, and the volume fractions of both the solvent and crystalline domains and calculated using complex free volume theory (see Section 2.5). Equation (1) is valid from the center of the polymer particle (*r* = 0) to the polymer particle–solvent interface (*r* = *R*(*t*)).

The boundary and initial conditions are as follows:

(2) $$\begin{array}{c} 0\leq r\leq R\left(t\right),\;t=0\;\;\;\emptyset_{s}=0\\ r=0,\;t>0\;\;\;\;\frac{\partial \emptyset_{s}}{\partial r}=0\\ r=R\left(t\right),\;t>0\;\;\;\;\;\emptyset_{s}=\emptyset_{s,i} \end{array}$$

where ∅*_s,i_* is the volume fraction of the solvent at the polymer–solvent interface, determined by solving a modified form of the Flory–Rehner equation [33]. The classical theory was adapted to account for the concentration dependence of the molecular weight between entanglements (*M_e_*) in concentrated polymer solutions [34,35], resulting in the following expression:

(3) $$\ln\left(\emptyset_{s,i}\right)+\left(1-\emptyset_{s,i}\right)+\chi_{12}{\left(1-\emptyset_{s,i}\right)}^{2}+V_{1}\rho_{p}\left(\frac{{(1-\emptyset }_{s,i})}{{{(M}_{e})}_{melt}}-\frac{2}{M}\right)\left({\left(1-\emptyset_{s,i}\right)}^{1/3}-\frac{\left(1-\emptyset_{s,i}\right)}{2}\right)=0$$

$\chi_{12}$ is the Flory–Huggins solvent–polymer interaction parameter, *V*_1_ is the molar volume of the solvent (see Table 1), $\rho_{p}$ is the polymer density, and *M* is the polymer molecular weight. $\chi_{12}$ is estimated using the Hansen solubility parameters [36,37,38,39]:

(4) $$\chi_{12}=\frac{V_{1}}{RT}\left({\left(\delta_{ds}-\delta_{dp}\right)}^{2}+0.25{\left(\delta_{ps}-\delta_{pp}\right)}^{2}+0.25{\left(\delta_{hs}-\delta_{hp}\right)}^{2}\right)+\beta$$

*R* is the gas constant and *T* the temperature (K). $\delta_{ds},\;\delta_{ps}$, and $\delta_{hs}$ are the Hansen solubility parameters of the solvent, and $\delta_{dp},\;\delta_{pp}$, and $\delta_{hp}$ are those of the polymer; Hansen solubility parameters are listed in Table 1. β is the entropic correction term, which is often approximated as 0.34 [38,39]. Using the values from Table 1, $\chi_{12}$ for decane and decalin was calculated via Equation (4) and is reported in Table 2. The corresponding ∅*_s,i_* values at various temperatures were calculated using Equation (3), and the results are summarized in Table 2.

### 2.2. Polymer Decrystallization

The transformation of crystalline domains into amorphous regions is described by a second-order rate equation, as adopted from prior studies [40].

(5) $$\frac{\partial \emptyset_{p,c}}{\partial t}=-k_{dec}\emptyset_{p,c}\emptyset_{s}$$

where ∅*_p,c_* is the volume fraction of crystalline domains within the polymer, and *k_dec_* is the decrystallization constant, which captures the rate at which crystalline regions are disrupted and converted into amorphous domains upon exposure to solvent. The crystalline domains of the polymer at *t* = 0 are assumed to be uniformly distributed throughout its volume.

### 2.3. Diffusion of Polymer Chains in Boundary Layer

The diffusion of polymer chains in the gel-like boundary layer is obtained as follows:

(6a) $$\frac{\partial \emptyset_{p,a}}{\partial t}=\frac{1}{r^{2}}\frac{\partial }{\partial r}\left(r^{2}D_{p}\frac{\partial \emptyset_{p,a}}{\partial r}\right)-\frac{dR}{dt}\frac{\partial \emptyset_{p,a}}{\partial r}$$

where ∅*_p,a_* is the volume fraction of amorphous polymer domains, *D_p_* is the diffusion coefficient of polymer chains in the solvent, and $\frac{dR}{dt}$ is the change in polymer particle radius over time, as determined in Section 2.4.

The model incorporates a delay known as the induction time (*t_ind_*), which is often observed between the initial polymer–solvent contact and the start of chain dissolution in semicrystalline polymers [40,41,42,43]. After this period, disentangled polymer chains diffuse through a gel-like boundary layer into the solvent (see Figure 1). The thickness of this layer (*δ*) is assumed constant, based on high-Reynolds-number flow conditions [44]. Prior studies report *δ* values around 50 μm for similar systems (e.g., tablets with 8 mm diameter and 5 mm thickness, *D*) [44], corresponding to *δ*/*D* ≈ 0.01. Accordingly, a *δ*/*r* ratio of 0.02 is used here and held constant across all solvents and temperatures.

Equation (6a) is applicable within the gel-like boundary layer, extending from the polymer–gel interface (*r* = *R*^+^(*t*)) to the gel–solvent interface (*r* = *R*^+^(*t*) + *δ*); see Figure 1). The corresponding boundary and initial conditions are given below:

(6b) $$R^{+}\left(t\right)\leq r\leq R^{+}\left(t\right)+\delta ,\;\;\;\;t=0,\;\;\;\emptyset_{p,a}=0$$

(6c) $$r=R^{+}\left(t\right),\;\;\;\;0<t<t_{ind},\;\;\;\frac{\partial \emptyset_{p,a}}{\partial r}=0$$

(6d) $$r=R^{+}\left(t\right),\;\;\;\;t_{ind}\leq t\;\;\;D_{p}\frac{\partial \emptyset_{p,a}}{\partial r}=-r_{dis}$$

(6e) $$r=R^{+}\left(t\right)+\delta ,\;\;\;\;\emptyset_{p,a}=0$$

where *r_dis_* is the disentanglement rate, which captures the rate at which polymer chains disengage from the entangled network and become available for diffusion into the surrounding solvent. According to the equations above, before the induction time, no flux of disentangled amorphous chains enters the gel-like boundary layer; afterward, the flux is directly governed by the disentanglement rate.

### 2.4. Change in Polymer Particle Radius

As solvent diffuses into the polymer and dissolution progresses, the radius of the polymer particle changes over time. This change can be determined by applying a mass balance on the polymer particle. Prior to the induction time, no dissolution takes place, and therefore the polymer mass remains constant, i.e., $\left(m_{t=0}=m_{t}\right)$. At time t=0, the polymer mass is given by $m_{t=0}=\rho \frac{4}{3}\pi {R_{1}}^{3}$, where ρ is the polymer density, and $R_{1}$ is the initial polymer particle radius. At time t, the mass can also be expressed as $m_{t}=\rho \left(\sum_{i=1}^{N}(1-{\emptyset_{s}}_{i}){dv}_{i}\right)\frac{4}{3}\pi {R_{t}}^{3}$, where $R_{t}$ is the particle radius at time t. Here, i represents a radial node, N is the total number of radial nodes, ${\emptyset_{s}}_{i}$ is the local solvent volume fraction at node i, and ${dv}_{i}$ is the differential volume at node i. Since the sum of the polymer and solvent volume fractions at each node must equal 1, the term $\sum_{i=1}^{N}(1-{\emptyset_{s}}_{i}){dv}_{i}$ accounts for the total polymer volume fraction. Rearranging the mass balance yields the following equation for the polymer particle radius over time:

(7) $${0<t<t_{ind},\;\;\;\;\;\;\;\;R}_{t}={\left(\frac{{R_{1}}^{3}}{\sum_{i=1}^{N}(1-{\emptyset_{s}}_{i}){dv}_{i}}\right)}^{1/3}$$

After the induction time, polymer dissolution begins, resulting in the presence of both dissolved and undissolved polymers at any given time. Consequently, the initial polymer mass ($m_{t=0}$) is equal to the sum of the undissolved polymer mass (${\left(m_{un}\right)}_{t}$) and the dissolved polymer mass (${\left(m_{diss}\right)}_{t}$), expressed as follows:

(8) $$m_{t=0}={\left(m_{un}\right)}_{t}+{\left(m_{diss}\right)}_{t}$$

The undissolved polymer mass at time t is given by the following:

(9) $${\left(m_{un}\right)}_{t}=\rho \left(\sum_{i=1}^{N}(1-{\emptyset_{s}}_{i}){dv}_{i}\right)\frac{4}{3}\pi {R_{t}}^{3}$$

The mass of dissolved polymer is obtained by integrating the flux of disentangled chains over time:

(10) $${\left(m_{diss}\right)}_{t}=\rho \sum_{j=1}^{j-1}r_{dis}4\pi {R_{j}}^{2}dt$$

where j refers to the time steps, dt is the time increment between steps j and j+1, and $R_{j}$ is the polymer particle radius at each time step. Rearranging the mass balance gives the equation for polymer particle radius over time after the induction time:

(11) $${t_{ind}\leq t,\;\;\;\;\;\;\;\;R}_{t}={\left(\frac{{R_{1}}^{3}-3\sum_{j=1}^{j-1}r_{dis}{R_{j}}^{2}dt}{\sum_{i=1}^{N}(1-{\emptyset_{s}}_{i}){dv}_{i}}\right)}^{1/3}$$

Accordingly, the change in polymer particle size over time can be approximated using the following finite difference expression:

(12) $${\left(\frac{dR}{dt}\right)}_{j}\approx \frac{R_{j}-R_{j-1}}{dt}$$

### 2.5. Diffusion Coefficient of Solvent in Polymer

Defining an appropriate expression for solvent diffusivity within the polymer matrix is critical for accurately modeling the dissolution process [45,46]. Experimental observations indicate that solvent diffusivity increases during dissolution, suggesting its dependence on temperature, the polymer–solvent type, and the volume fraction of the solvent within the polymer [46]. To account for these dependencies, the Vrentas–Duda free volume theory is employed in this study to describe solvent diffusivity in the polymer [47,48]. The Vrentas–Duda equation has demonstrated strong capability in capturing the complex diffusion behavior of solvents in semicrystalline polymers:

(13) $$D_{s}=D_{sa}[(1-2\chi_{12}\emptyset_{sa}){\left(1-\emptyset_{sa}\right)}^{2}](1-\emptyset_{p,c})$$

*D_sa_* is the self-diffusion coefficient of the solvent in the amorphous phase, and ∅*_sa_* is the volume fraction of the solvent in the amorphous phase. Equation (14) defines *D_sa_* as follows [47]:

(14) $$D_{sa}=[D_{01}exp(\frac{-E^{*}}{RT})exp(\frac{w_{sa}{V_{s}}^{*}+(1-w_{sa}){V_{p}}^{*}\xi }{V_{{FH}_{a}}/\gamma })]$$

*D*_01_ is the pre-exponential factor; *ω_sa_* is the weight fraction of the solvent in the polymer amorphous phase; V_s_^*^ and V_p_^*^ are the critical hole free volumes of the solvent and polymer, respectively; *ξ* is the ratio of the solvent and polymer jumping units; E^*^ is the energy required for a solvent molecule to overcome the attractive forces when moving through the mixture (most cases = 0); and *V_FHa_* is the sum of the hole free volumes of the solvent and polymer in the amorphous phase [47]. The weight fraction of the solvent in the amorphous phase (*ω_sa_*) is obtained using Equation (15):

(15) $$\omega_{sa}=\frac{1}{1+\frac{\emptyset_{p,a}\times \rho_{p,a}}{\emptyset_{s\times }\rho_{s}}}$$

where ∅*_p,a_* is the volume fraction of the amorphous polymer, *ρ_p,a_* is the density of the amorphous polymer (assumed, for simplicity, to equal the overall polymer density), and *ρ_s_* is the density of the solvent. The total hole free volume of the solvent and polymer in the amorphous phase (*V_FHa_*) is determined as follows:

(16) $$V_{{FH}_{a}}/\gamma =w_{sa}\frac{K_{11}}{\gamma_{1}}\left[\left(K_{21}-T_{gs}\right)+T\right]+(1-w_{1a})\frac{K_{12}}{\gamma_{2}}\left[\left(K_{22}-T_{gp}\right)+T\right]$$

where *K*_11_ and *K*_21_ are the basic free volume parameters related to the solvent, while *K*_22_ and *K*_12_ correspond to the polymer (PP) and are calculated based on the WLF (Williams, Landel, and Ferry) equation constants [47]. *T_gs_* and *T_gp_* are the glass transition temperatures of the solvent and polymer, respectively. The free volume parameters of the solvents (decane and decalin) and the polymer (PP) considered in this study are listed in Table 1.

### 2.6. Diffusion Coefficient of Disentangled Polymer Chains in Solvent

To estimate *D_p_*, it is assumed that disentangled polymer chains undergo Brownian motion within the gel-like boundary layer [40]. Accordingly, their diffusion coefficient in various solvents can be related to solvent viscosity via the Stokes–Einstein equation [40]:

(17) $$D_{p}=\frac{K_{B}\times T}{\sqrt{6}\times \pi \times {ŋ}_{s}\times R_{g}}$$

where *K_B_* is the Boltzmann constant, *ŋ_s_* is the solvent viscosity, and *R_g_* is the radius of gyration for linear ideal polymer chains. The parameter values used in Equation (17), along with the calculated diffusion coefficients of PP in various solvents at different temperatures, are listed in Table 2.

### 2.7. Solving the Governing Equations

To solve the governing equations, the moving boundary problem is reformulated as a fixed boundary problem using dimensionless spatial coordinates through the Landau transformation, defined as follows:

(18) $$\xi =\frac{r}{R(t)},\;\;\;\;\;\;\;\begin{array}{c} 0<r<R(t)\\ 0<\xi <1 \end{array}$$

where *ξ* represents the dimensionless spatial coordinate within the polymer. The derivatives of *ξ* with respect to time *t* and position *r* are given by the following:

(19) $$\frac{\partial \xi }{\partial t}=\frac{-\xi }{R}\frac{dR}{dt},\;\;\frac{\partial \xi }{\partial r}=\frac{1}{R}$$

Let *f* be a representative variable for ∅*_s_*, ∅_*p*,*c*_, and ∅_*p*,*a*_, each dependent on radial position *r* and time *t*. By applying the chain rule, the derivatives in the original moving-boundary (*mov*) and fixed-boundary (*fix*) coordinate systems are related as follows:

(20) $${\left(\frac{\partial f}{\partial t}\right)}_{mov}={\left(\frac{\partial f}{\partial t}\right)}_{fix}\frac{\partial t}{\partial t}+{\left(\frac{\partial f}{\partial \xi }\right)}_{fix}\frac{\partial \xi }{\partial t}={\left(\frac{\partial f}{\partial t}\right)}_{fix}-{\frac{-\xi }{R}\frac{dR}{dt}\left(\frac{\partial f}{\partial \xi }\right)}_{fix}$$

(21) $${\left(\frac{\partial f}{\partial r}\right)}_{mov}={\left(\frac{\partial f}{\partial t}\right)}_{fix}\frac{\partial t}{\partial r}+{\left(\frac{\partial f}{\partial \xi }\right)}_{fix}\frac{\partial \xi }{\partial r}=\frac{1}{R}{\left(\frac{\partial f}{\partial \xi }\right)}_{fix}$$

(22) $${\left(\frac{\partial^{2}f}{\partial r^{2}}\right)}_{mov}={\frac{\partial }{\partial r}\left(\frac{\partial f}{\partial r}\right)}_{mov}=\frac{\partial }{\partial r}\left(\frac{1}{R}{\left(\frac{\partial f}{\partial \xi }\right)}_{fix}\right)=\frac{\partial \left(\frac{1}{R}{\left(\frac{\partial f}{\partial \xi }\right)}_{fix}\right)}{\partial t}\frac{\partial t}{\partial r}+\frac{\partial \left(\frac{1}{R}{\left(\frac{\partial f}{\partial \xi }\right)}_{fix}\right)}{\partial \xi }\frac{\partial \xi }{\partial r}=\frac{1}{R^{2}}{\left(\frac{\partial^{2}f}{\partial \xi^{2}}\right)}_{fix}$$

Using Equations (20)–(22), the governing Equation (1) and Equations (5) and (6a) along with their corresponding initial and boundary conditions are transformed into the following:

(23) $$\frac{\partial \emptyset_{s}}{\partial t}=\frac{\xi }{R}\frac{dR}{dt}\;\left(\frac{\partial \emptyset_{s}}{\partial \xi }\right)+\frac{2D_{s}}{\xi }\frac{1}{R^{2}}\left(\frac{\partial \emptyset_{s}}{\partial \xi }\right)+\frac{D_{s}}{R^{2}}\left(\frac{\partial^{2}\emptyset_{s}}{\partial \xi^{2}}\right)\;\;\;\;\;\;\;\;\;\;\;\;\begin{array}{c} t=0,\;\;\;\emptyset_{s}=0\\ \xi =0,\;t>0\;\;\;\frac{\partial \emptyset_{s}}{\partial \xi_{1}}=0\\ \xi =1,\;t>0\;\;\;\emptyset_{s}=\emptyset_{s,i} \end{array}$$

(24) $$\frac{\partial \emptyset_{p,c}}{\partial t}=\frac{\xi }{R}\frac{dR}{dt}\;\left(\frac{\partial \emptyset_{p,c}}{\partial \xi }\right)-k_{dec}\emptyset_{p,c}\emptyset_{s}\;\;\;\;\;\;\;\;\;\;\;\;\;t=0,\;\;\;\emptyset_{p,c}=d_{c}$$

To solve the diffusion equation for polymer chains in the gel-like boundary layer, the radial coordinate is transformed to *r*_2_, defined as follows:

(25) $${r_{2}}_{i}=R\left(t\right)+(i-2)dr_{2}$$

where *R*(*t*) is the polymer particle radius at time *t*, *i* indexes the radial nodes in the gel layer (1 ≤ *i* ≤ *N*_2_), and *N*_2_ is the total number of nodes in this layer. The node spacing is defined as *dr*_2_ = *δ*/(*N*_2_ − 1). With this substitution, the diffusion equation for polymer chains within the gel-like boundary layer simplifies to the following:

(26) $$\frac{\partial \emptyset_{p,a}}{\partial t}=\frac{2D_{p}}{r_{2}}\frac{\partial \emptyset_{p,a}}{\partial r_{2}}+D_{p}\frac{\partial^{2}\emptyset_{p,a}}{\partial {r_{2}}^{2}}-\frac{dR}{dt}\frac{\partial \emptyset_{p,a}}{\partial r_{2}}$$

By applying the Finite Difference Method (FDM) to the derivatives in Equations (23) and (24), the following is obtained:

(27) $$\begin{array}{l} 1<i<N_{1}:\;\frac{{\emptyset_{s}}_{i}^{j+1}-{\emptyset_{s}}_{i}^{j}}{dt}\;\;\;\;\;\;\;\;\;\;\;\;\;\;\;\;\;\;\;\;\;\;\;\;\;\;\;\;\;\;\;\;\;\;\;\;\;\;\;\;\;\;\;\;\;\;\;\;\;\;\;\;\;\;\;\;\;t=0,\;\;\;{\emptyset_{s}}_{i}=0\\ =\left(\frac{\xi }{R^{j}}{\left(\frac{dR}{dt}\right)}^{j}+\frac{2D_{s}}{\xi }\frac{1}{{R^{j}}^{2}}\right)\left(\frac{{\emptyset_{s}}_{i+1}^{j+1}-{\emptyset_{s}}_{i-1}^{j+1}}{2d\xi }\right)\;\;\;\;\;\;\;\;\;\;\;\;\;\;\;\;\;\;\;\;\;\;\;i=1:\;\frac{{\emptyset_{s}}_{i+1}^{j+1}-{\emptyset_{s}}_{i}^{j+1}}{d\xi }=0\\ +\frac{D_{s}}{{R^{j}}^{2}}\frac{{\emptyset_{s}}_{i+1}^{j+1}-2{\emptyset_{s}}_{i}^{j+1}+{\emptyset_{s}}_{i-1}^{j+1}}{{d\xi }^{2}}\;\;\;\;\;\;\;\;\;\;\;\;\;\;\;\;\;\;\;\;\;\;\;\;\;\;\;\;\;\;\;\;\;\;\;\;\;\;\;\;\;\;\;\;\;\;\;\;\;\;i=N_{1}:\;{\emptyset_{s}}_{i}^{j+1}=\emptyset_{s,i} \end{array}$$

(28) $$\begin{array}{l} 1\leq i\leq N_{1}:\;\frac{{\emptyset_{p,c}}_{i}^{j+1}-{\emptyset_{p,c}}_{i}^{j}}{dt}\;\;\;\\ =\frac{\xi }{R^{j}}{\left(\frac{dR}{dt}\right)}^{j}\frac{{\emptyset_{p,c}}_{i+1}^{j+1}-{\emptyset_{p,c}}_{i}^{j+1}}{d\xi }\;\;\;\;\;\;\;\;\;\;\;\;\;\;\;\;\;\;\;\;\;\;\;\;\;\;\;\;\;t=0,\;\;\;{\emptyset_{p,c}}_{i}=d_{c}\\ -k_{dec}{\emptyset_{p,c}}_{i}^{j+1}{\emptyset_{s}}_{i}^{j+1} \end{array}$$

Applying the FDM to the derivatives in Equation (26) yields the following:

(29) $$\begin{array}{l} 1<i<N_{2}:\;\frac{{\emptyset_{p,a}}_{i}^{j+1}-{\emptyset_{p,a}}_{i}^{j}}{dt}\;\;\;\;\;\;\;\;\;\;\;\;\;\;\;\;\;\;\;\;\;\;\;\;\;\;\;\;\;\;\;\;\;\;\;\;\;\;\;\;\;0\leq t<t_{ind},\;\;\;\emptyset_{p,a}=0\\ =\frac{2D_{p}}{{r_{2}}_{i}}-{\left(\frac{dR}{dt}\right)}^{j}\left(\frac{{\emptyset_{p,a}}_{i+1}^{j+1}-{\emptyset_{p,a}}_{i-1}^{j+1}}{2dr_{2}}\right)\;\;\;\;\;\;\;\;\;\;\;\;\;\;\;\;\;\;\;\;\;\;\;\;\;i=1,t_{ind}<t:\;-D_{p}\frac{{\emptyset_{p,a}}_{i+1}^{j+1}-{\emptyset_{p,a}}_{i}^{j+1}}{dr_{2}}=r_{dis}\\ +D_{p}\frac{{\emptyset_{p,a}}_{i+1}^{j+1}-2{\emptyset_{p,a}}_{i}^{j+1}+{\emptyset_{p,a}}_{i-1}^{j+1}}{{dr_{2}}^{2}}\;\;\;\;\;\;\;\;\;\;\;\;\;\;\;\;\;\;\;\;\;\;\;\;\;\;\;\;\;\;\;\;\;\;\;i=N_{2}:\;\emptyset_{p,a}=0 \end{array}$$

The equations were solved in MATLAB (R2024b) using a time step of 0.1 s, with 40 spatial intervals within the polymer particle and 20 intervals in the gel-like boundary layer.

### 2.8. Model Inputs, Parameters, and Predictions

The polymer dissolution kinetic model has the following experimental inputs: temperature (*T*), initial particle radius (*R*_1_), initial degree of crystallinity (*d_c_*), and polymer molecular weight (*M*).

Several parameters were sourced from the literature, including solvent viscosity (*ƞ_s_*), solvent molar volume (*V*_1_), Hansen solubility parameters for the solvent and polymer (*δ_d_*, *δ_p_*, and *δ_h_*), and boundary layer thickness (*δ*). The Flory–Huggins interaction parameter (*χ*_12_) was calculated from the Hansen solubility parameters using Equation (4), which then informed the calculation of the solvent volume fraction at the polymer–solvent interface (∅*_s,i_*) via the Flory–Rehner equation (Equation (3)). The diffusion coefficient of polymer chains in the solvent (*D_p_*) was estimated using the Stokes–Einstein relation (Equation (17)). The parameter values used in the model calculations are compiled in Table 1 and Table 2.

The key fitted parameters in the dissolution model are the decrystallization rate constant (*K_dec_*) and polymer chain disentanglement rate (*r_dis_*). These were determined by simultaneously fitting two independent experimental datasets, the dissolved polymer amount over time and time required for full decrystallization, and minimizing the deviation between experimental data and model predictions. The induction time (*t_ind_*) was also obtained from model fitting.

The model outputs include the temporal evolution of the polymer particle radius, *R*(*t*), and the spatiotemporal profiles within the dissolving particle for the crystalline and amorphous polymer volume fractions (∅_*p*,*c*_, ∅_*p*,*a*_), solvent volume fraction (∅*_s_*), and solvent volume fraction within the amorphous phase (∅_*s*,*a*_). Additionally, the model computes the solvent diffusivity in the polymer (*D_s_*) and the solvent self-diffusion coefficient in the amorphous phase (*D_sa_*), both of which vary spatially and temporally due to their dependence on local volume fractions.

### 2.9. Parameters Specific to Polypropylene–Solvent Systems

The polymer dissolution kinetic model and governing equations presented above are formulated within a general framework that captures solvent diffusion, polymer decrystallization, chain disentanglement, and particle radius evolution, making it applicable to a wide range of semicrystalline polymers. In this work, the developed model is applied specifically to the dissolution of PP in decane and decalin, at 130, 150, and 170 °C. The specific properties of the polymer and solvents used in this study are compiled in Table 1 and Table 2. Table 1 summarizes the Hansen solubility parameters and the solvent and polymer free volume parameters required for the Vrentas–Duda solvent diffusivity equation, while Table 2 presents the Flory–Huggins interaction parameters, solvent volume fractions at the polymer–solvent interface, and the calculated diffusion coefficients of polymer chains in the solvents across the studied temperatures.

**Table 1:** Hansen solubility parameters (J/cm^3^)^1/2^, solvent molar volumes (cm^3^/mol) [49], and free volume parameters of solvents and PP [48].

Material	*δ_d_*	*δ_p_*	*δ_h_*	*V*_1_	V_S_^*^, or V_p_^*^ (cm^3^/g)	*K*_11_/*γ*_1_, or *K*_12_/*γ*_2_ (cm^3^/g.k) (×10^−4^)	*K*_21_*-T_gs_*, or *K*_22_*-T_gp_* (K)	*D*_01_ (cm^2^/s) (×10^−4^)	E* (cal/mol)	*ξ ^i^*
PP	18.1	0	1	-	1.005	5.02	−205.4	-	-	-
Decane	15.7	0	0	195.9	1.082	12.2	−55.14	5.22	0	0.5
Decalin	18.8	0	0	156.9	0.928	10.9	−100.5	7.08	0	0.43

(*^i^*) estimated based on its correlation with the solvent’s molar volume at 0 K [48].

**Table 2:** Thermodynamic and transport parameters for PP dissolution, including Flory–Huggins interaction parameter, solvent volume fraction at PP–solvent interface (∅*_s,i_*), and diffusion coefficients of PP chains in solvents at different temperatures (PP: $\rho_{p}$ = 0.905 g/cm^3^; (*M_e_*)*_melt_* = 7700 g/mol [50,51]; *M* = 170,000 g/mol [26]; *K_B_* = 1.38 × 10^−23^ J/K).

Solvent	Temperature (°C)	$\chi_{12}$	∅*_s,i_*	*D_p_^i^* (m^2^/s) (×10^−11^)
Decane	130	0.6913	0.887	6.07
	150	0.6747	0.89	6.37
	170	0.6596	0.8912	6.67
Decalin	130	0.3746	0.9144	2.412
	150	0.3730	0.9148	2.532
	170	0.3715	0.9153	2.652

(*^i^*) To calculate *D_p_* using Equation (17), the radius of gyration (*R_g_*) of PP was taken as $0.34\;\sqrt{M}$ (Å) [52]. The viscosities of decane and decalin were considered as 0.85 [53] and 2.138 [54] mPa.s, respectively, with the decalin value corresponding to a 50/50 *cis*-/*trans*-decalin mixture. The resulting *R_g_* was calculated as 14.018 nm.

## 3. Experiments

### 3.1. Polymer and Solvents

Polypropylene pellets with a density of 0.90–0.92 g/cm^3^ and a melting range of 160–170 °C, produced by Braskem S.A. and supplied by Braskem America, Inc. (Philadelphia, PA, USA), were used in this study. PP had a weight-average molar mass of approximately 170,000 g/mol. The equivalent spherical radius (*R*_1_) was calculated from the average pellet volume, determined by measuring the mass of 100 pellets and applying the known density. This approach, used in similar studies [31,32], simplifies the complex pellet geometry while preserving volume equivalence for modeling. The equivalent spherical radius (*R*_1_) was approximately 1.8 mm.

The following solvents were used in this study: n-decane (99.0%, pure), with MW = 142.29 g/mol, MP = −30 °C, density of 0.730 g/mL, and BP = 172–174 °C (Thermo Fisher Scientific Inc., Boston, MA, USA), and decahydronaphthalene (cis/trans mixture, ≥99.0%, pure), also known as decalin, with MW = 138.25 g/mol, MP = −31 °C, density of 0.890 g/mL, and BP = 190 °C (TCI America, Boston, MA, USA).

### 3.2. Decrystallization and Dissolution of PP Pellets

Decrystallization is a critical step in the dissolution of semicrystalline polymers such as PP, since crystalline domains restrict solvent diffusion and polymer chain mobility. To evaluate the role of decrystallization in PP dissolution kinetics, we conducted decrystallization experiments in decalin and decane at 130, 150, and 170 °C (±1 °C temperature fluctuation). These solvents and temperatures were selected based on experimental data for the dissolved fraction of PP pellets [26].

**Figure 2:**
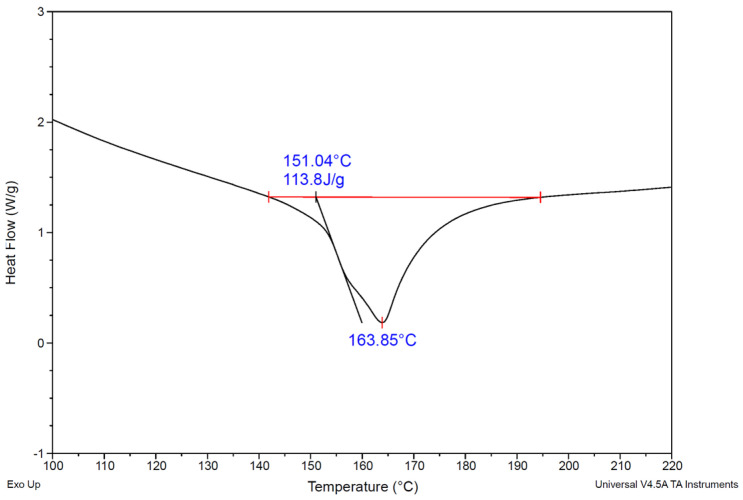
DSC thermograms of polypropylene, showing an initial melt onset at 151.04 °C, a peak melt temperature of 163.85 °C, and an enthalpy of fusion of 113.8 J/g.

The degree of crystallinity of the PP pellets used in this study, prior to any decrystallization, was determined via DSC using a TA Instruments SDT Q600. PP pellets were crimped into Tzero aluminum pans (TA Instruments, New Castle, DE, USA, P/N: 901683.901) and lids (TA Instruments, P/N:901671.901). Nitrogen was sparged through the system at a rate of 100 mL/min to avoid the oxidation of the polymer. Samples were equilibrated at 25 °C, then heated to 300 °C at a rate of 10 °C/min, followed by cooling to 25 °C at the same rate. The degree of crystallinity was determined from the first heating cycle using the integrated peak with a linear baseline; the same value was obtained using the sigmoidal horizontal baseline (see Figure 2). The average crystallinity of the PP pellets was 55%, which is the same (within the ±1% experimental uncertainty associated with DSC measurements) as the 54% value in the study that reported the amount of PP dissolved [26]. Although that study did not describe the procedure used to determine the initial crystallinity of PP, the same authors previously used a linear baseline for both melting and crystallization peaks [27]. Hence, applying the linear baseline here enables a direct comparison of the results.

**Figure 3:**
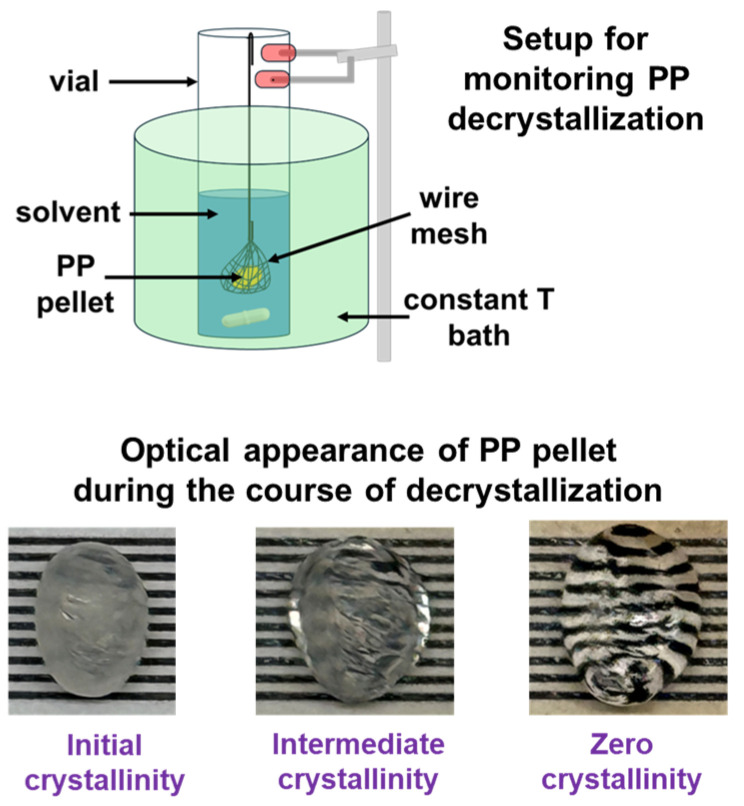
Experimental setup for monitoring PP decrystallization and optical appearance of PP pellet during course of decrystallization.

To initiate the decrystallization process, PP pellets were placed inside a stainless-steel wire mesh (cage-like holder) to ensure stable positioning and uniform solvent contact. The mesh containing the polymer was fully immersed in a 10 mL vial of preheated solvent, and the system was stirred at 200 rpm while being maintained at a target temperature of 130, 150, or 170 °C. The wire mesh prevented the polymer from adhering to the vial walls or settling at the bottom while also allowing for unrestricted solvent access to all particle sides. The decrystallization process started immediately upon immersion. The progress of decrystallization was monitored visually for 6 replicates at each temperature, with the polymer’s transition to complete transparency (signifying loss of crystalline domains) serving as the key qualitative indicator of complete decrystallization (see Figure 3). The average full decrystallization times along with the standard deviation are reported.

The time-dependent fraction of dissolved PP (mass dissolved relative to initial mass) in decalin and decane at 130, 150, and 170 °C was obtained from a publication on the isothermal dissolution of PP pellets [26] and used here, together with the decrystallization data discussed above, as a quantitative benchmark to evaluate the accuracy of the model in predicting dissolution kinetics.

## 4. Results and Discussion

### 4.1. Experimental Data on PP Decrystallization and Dissolution Kinetics

Experimental data on the decrystallization and dissolution kinetics of spherical PP pellets in decalin and decane are discussed here, with a focus on the effects of solvent type and temperature.

**Figure 4:**
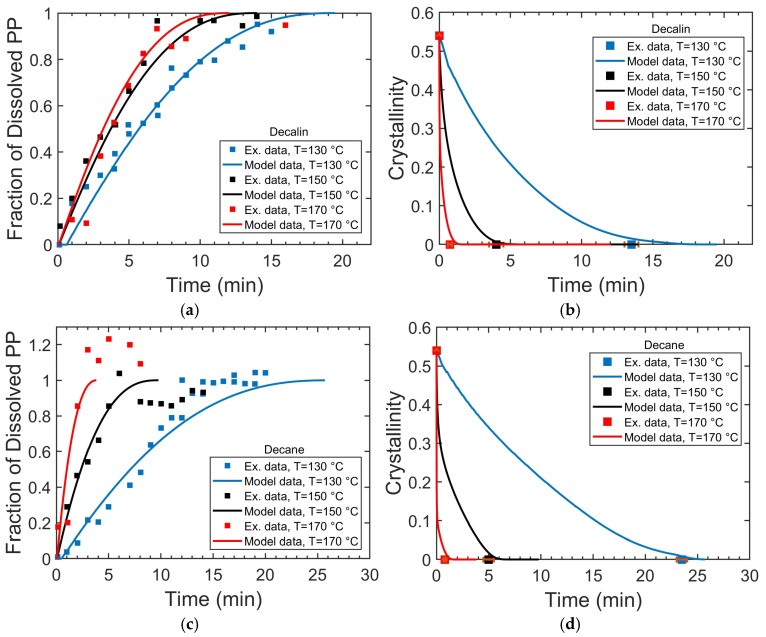
The experimental results (dots) and model fits (solid lines) for spherical PP pellets in decalin and decane at 130, 150, and 170 °C. Plots (**a**,**b**) present the dissolved polymer fraction and crystallinity evolution, respectively, in decalin, while plots (**c**,**d**) show the corresponding results in decane.

The dissolution kinetics of PP pellets followed a consistent trend across all solvents and temperatures: an initially rapid dissolution that gradually slows down, approaching a plateau as the particle becomes smaller and solvent diffusion pathways shorten (see Figure 4). The pellets fully decrystallize before dissolving completely, indicating a multi-step process involving initial solvent diffusion and crystalline disruption, followed by chain disentanglement and diffusion into the solvent [40].

Increasing temperature reduces the time required for both the complete decrystallization and dissolution of PP pellets. At 130 °C, the solvent type strongly influences the dissolution kinetics, with pellets in decalin decrystallizing in ~13 min and dissolving completely in ~19 min, compared to ~23 min and ~27 min in decane. However, at higher temperatures (e.g., 170 °C), which are near the melting point of PP (166.5 °C [26]), the pellets decrystallize and dissolve rapidly in both solvents, indicating that solvent choice becomes less critical under these conditions.

We note that while raising temperature accelerates dissolution, elevated temperatures may cause polymer degradation, solvent losses, and increased energy consumption [55,56]. Since temperature influences both the overall kinetics and the rate-limiting step (decrystallization or chain disentanglement), the temperature and solvent should be selected in conjunction.

In the case of PP dissolution kinetics, the influence of solvent evolves with temperature. At the lower end of the temperature range considered here (130 °C), decalin outperforms decane, and the overall dissolution rate is primarily limited by the disruption of crystalline domains, as evidenced by the close correspondence between the full decrystallization times (~13 min in decalin, ~23 min in decane; Figure 4b,d) and the complete dissolution times (~19 min in decalin, ~27 min in decane; Figure 4a,c). At elevated temperatures (150 and 170 °C), crystalline regions are rapidly disrupted in both solvents, shifting the rate-limiting step to polymer chain disentanglement, a process in which decane proves more effective, resulting in faster complete dissolution. Thus, increasing temperature not only accelerates the overall dissolution rate but also alters the dominant dissolution mechanism and the relative effectiveness of different solvents.

Blackadder & Le Poidevin [25] reported that PP at 120 °C dissolved faster in n-alkanes than in aromatics or naphthenics of comparable molar volume and attributed this to the greater molecular flexibility of n-alkanes facilitating diffusivity in the polymer matrix [25]. This is consistent with the observation that at 150 and 170 °C, decalin (naphthenic solvent) is less effective than decane (linear alkane) despite its lower molar volume. Very recently, Ferchichi et al. [27] reported that the presence of a solvent lowers the melting point (decrystallization temperature) of PP to approximately 100 °C in decalin and 123 °C in decane. Based on this, they ranked decalin as more effective than decane for dissolving and decrystallizing PP and proposed decrystallization temperature as a qualitative indicator of a solvent’s ability to dissolve polypropylene [27].

### 4.2. Validation of Model That Describes PP Decrystallization and Dissolution Kinetics

To evaluate the model’s validity and accuracy, its predictions were fitted to experimental data on PP decrystallization kinetics and dissolution kinetics under various conditions. As shown in Figure 4, the model fits the experimental results well for both the dissolved polymer fraction and the crystallinity time evolution across different temperatures and solvents. This validates the molecular-level processes and mechanisms described by the model and enables the use of the model to generate insights into the dissolution mechanisms and structural evolution of PP pellets. The key fitting parameters, the decrystallization constant (*K_dec_*) and disentanglement rate (*r_dis_*), were determined by fitting the model to experimental data across various solvents and temperatures. The visual determination of complete transparency introduces an uncertainty of ±30 s at 130 °C and 150 °C and ±10 s at 170 °C in both solvents. Sensitivity analysis shows that fitting with the upper-bound times (*t* + ∆*t*) decreases *K_dec_* by ~5–7% at 130 °C, 25–30% at 150 °C, and up to ~35–40% at 170 °C. *K_dec_* values near the melting region should be interpreted with this experimental uncertainty in mind.

A sensitivity analysis of *K_dec_* and *r_dis_* values on the times of full dissolution and decrystallization for PP in decalin at 150 °C indicates that the overall dissolution process is primarily governed by chain disentanglement, while the decrystallization kinetics have a more isolated effect (see Table 3). Increasing *r_dis_* at a constant *K_dec_* significantly accelerates dissolution but prolongs decrystallization only slightly. This inverse relationship arises because the rapid removal of amorphous regions limits solvent diffusion into the remaining crystalline domains, illustrating the coupled nature of amorphous chain mobility and crystal breakdown. In contrast, varying *K_dec_* at a fixed *r_dis_* directly controls the decrystallization rate but has a negligible impact on dissolution time. This indicates that, once sufficient decrystallization has taken place, the overall dissolution process becomes dominated by chain disentanglement.

### 4.3. Insights into Temperature- and Solvent-Controlled Mechanisms of PP Dissolution

**Figure 5:**
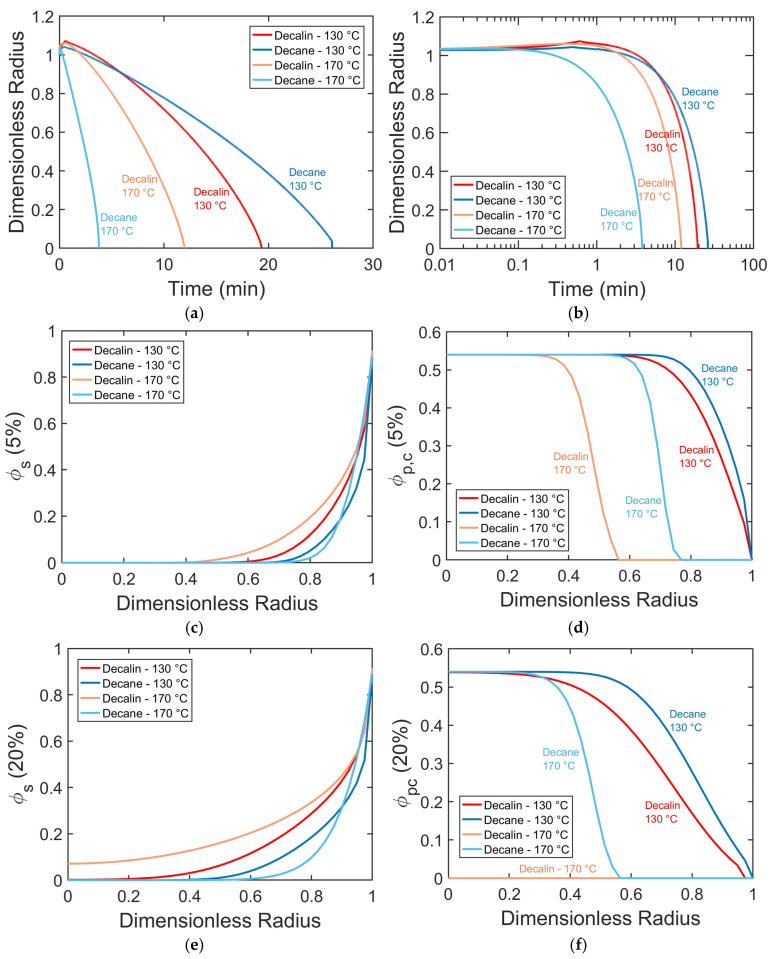
Dissolution kinetics of spherical PP pellets (radius 1.8 mm) in decalin and decane at 130 and 170 °C. (**a**,**b**) Dimensionless pellet radius vs. time in linear and logarithmic scales. (**c**,**d**) Radial profiles of volume fractions of solvent (∅*_s_*) and crystalline domains (∅*_p,c_*) in dissolving particle at 5% progression toward complete dissolution. (**e**,**f**) Corresponding profiles at 20% progression.

To elucidate the interplay between temperature and solvent type, Figure 5 presents model-derived predictions of PP pellet dissolution in decalin or decane at 130 and 170 °C. These results extend beyond the macroscopic kinetics shown in Figure 4 to reveal the time-dependent size of the dissolving particle and its composition as it changes from the particle center to its surface.

Increasing temperature accelerates PP dissolution in both solvents, with a more pronounced effect in decane (Figure 5a,b). Higher temperatures also reduce the degree of swelling by shortening the time PP pellets spend in the swollen state (*R*/*R*_1_ > 1; Figure 5a,b), due to accelerated crystalline disruption and chain disentanglement. The radial profiles of ∅*_p,c_* further confirm that increasing temperature enhances crystalline disruption across the particle, as the crystalline volume fraction decreases at both 5% and 20% progression toward complete dissolution (Figure 5d,f).

The solvent type strongly influences dissolution kinetics, and its effect is temperature-dependent. At 130 °C, decalin dissolves PP pellets faster than decane, as evidenced by its higher solvent volume fraction within the particle (Figure 5c,e) and earlier crystalline disruption (Figure 5d,f). At 170 °C, the trend reverses: decane dissolves PP pellets faster, although decalin shows higher ∅*_s_* within the particle and more rapid crystalline disruption (Figure 5c–f).

The relative influence of temperature and solvent thus depends on the specific conditions. At 130 °C, the solvent effect dominates, as the dissolution process is decrystallization-controlled (with most of the polymer interior still crystalline even at 20% progression (Figure 5f)). Under these conditions, decalin outperforms decane because its smaller molecular size promotes greater ∅*_s_* within the particle. In contrast, at 170 °C, the temperature effect dominates, as higher thermal energy accelerates complete polymer decrystallization (e.g., full decrystallization by 20% progression in decalin (Figure 5f)), shifting the rate-limiting step to chain disentanglement. This process is favored by solvents with a linear molecular shape, such as decane, which better matches the linear PP chains and facilitates chain mobility more effectively. This demonstrates a temperature-induced transition in the rate-limiting mechanism, from decrystallization-controlled kinetics at lower temperatures to disentanglement-controlled kinetics at higher temperatures.

### 4.4. Effect of Temperature on Decrystallization Rate Constant (K_dec_)

Evaluating the effect of temperature on *K_dec_* and *r_dis_* is essential, as these key parameters govern the rate at which a semicrystalline polymer transitions from an ordered solid to a fully dissolved state [28,30,40,57,58,59]. Temperature directly influences chain mobility [60,61,62] and diffusivity [63], both of polymer segments in solvent and of solvent molecules into the polymer matrix so that even small changes in temperature can significantly alter dissolution kinetics by accelerating crystallite disruption, chain disengagement, or both [57]. Understanding these temperature dependencies improves the prediction of PP dissolution kinetics while guiding process optimization to reduce the risk of polymer degradation.

When sufficient thermal energy is provided to overcome the lattice forces stabilizing crystalline domains, the ordered structure is disrupted, resulting in decrystallization/melting [64,65,66]. The sensitivity of crystalline domain disruption to temperature changes is reflected in polymer melting and crystallization kinetics that typically follow Arrhenius-type behavior [60,65,66,67]. The established Avrami kinetics of crystallization [68,69] provide compelling theoretical support for the temperature dependence of the decrystallization rate, revealing a fundamental symmetry between these complementary phase transformations. This behavior is consistent with the findings for cellulose decrystallization [57]. To quantitatively assess this temperature dependence, the obtained *K_dec_* values were fitted to an Arrhenius-type equation:

(30) $$K_{dec}=A\times exp(\frac{-E_{a}}{R\times T})$$

*A* is the pre-exponential factor (1/s) and *E_a_* the activation energy (kJ/mol).

**Figure 6:**
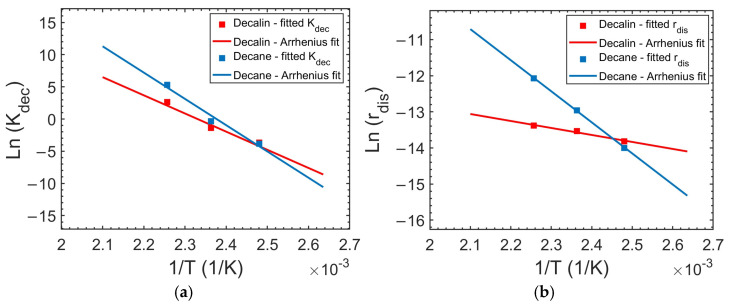
(**a**) *K_dec_* and (**b**) *r_dis_* values obtained by fitting the model to experimental data at 403, 423, and 443 K for decalin and decane; solid lines show the corresponding Arrhenius fits.

The Arrhenius plot of *K_dec_* is linear in both decalin and decane (Figure 6), validating the thermally activated nature of PP decrystallization. The Arrhenius regression yielded activation energies of 234 ± 0.5 kJ/mol (*A* = 3.56 × 10^28^ 1/s, *R*^2^ = 0.96) for PP decrystallization in decalin and 339 ± 0.4 kJ/mol (*A* = 1.36 × 10^42^ 1/s, *R*^2^ =0.97) in decane. These activation energies are about double of those reported for the crystallization of isotactic PP (*E_a_* ≈ 120–130 kJ/mol) [70], reflecting the additional energetic requirement for solvent molecules to disrupt the PP crystalline lattice. This interpretation aligns with DSC data [27] that show that while PP crystallizes at relatively low temperatures in decalin (~37 °C) and decane (~73 °C), decrystallization begins at significantly higher temperatures (~103 °C and ~123 °C, respectively) [27]. For comparison, the melting of polyethylene exhibits very high apparent activation energies (~485 kJ/mol near 140 °C) [67].

The lower *E_a_* obtained in decalin compared to decane highlights the influence of solvent properties on the energy barrier for polymer decrystallization. Decalin, a bicyclic, aromatic-like solvent, more efficiently disrupts crystalline domains, thereby lowering the energetic requirement. In contrast, while decane is less efficient at promoting decrystallization, requiring greater thermal input to achieve comparable rates, it leads to more rapid complete dissolution at elevated temperatures (150 and 170 °C, Figure 4). This illustrates that *E_a_* is modulated by solvent diffusivity, viscosity, and molecular-level interactions.

### 4.5. Effect of Temperature on Polymer Chain Disentanglement Rate (r_dis_)

Polymer disentanglement occurs when solvent molecules disrupt intermolecular interactions [71], primarily London dispersion forces [72], between polymer chains. As temperature increases, these interactions weaken [73], resulting in enhanced chain mobility, reduced flow resistance, and increased free volume [74]. This leads to a significant decrease in polymer melt viscosity with temperature [73,75]. While no single equation quantifies intermolecular interaction strength as a function of temperature, their net effect on macroscopic properties such as viscosity is captured by an Arrhenius-type relationship [76]. Since the rate of chain disentanglement depends on both intermolecular forces and chain mobility, each strongly temperature-dependent, the disengagement rate is also expected to exhibit Arrhenius behavior. The linearity of the Arrhenius plot for *r_dis_* (Figure 6) validates the use of the Arrhenius model to describe its temperature-driven behavior.

The Arrhenius fits for the disentanglement rate yielded activation energies of 16 ± 0.1 kJ/mol (*A* = 1.24 × 10^−4^ m/s, *R*^2^ = 0.98) in decalin and 71 ± 0.5 kJ/mol (*A* = 1.57 × 10^3^ m/s, *R*^2^ = 1.00) in decane. These activation energy values are comparable to activation energies for PP melt viscosity, which are reported in the range 39–88 kJ/mol, depending on shear stress [77]. This agreement supports the interpretation that solvent-driven disentanglement is governed by the same temperature-sensitive polymer chain relaxation processes that control viscous flow in molten PP.

The higher *E_a_* observed in decane suggests a more rapid increase in the PP disentanglement rate with temperature, helping to explain the reversal in overall dissolution performance at elevated temperatures. While decalin’s advantage lies in its superior ability to disrupt crystallites (see section: *Effect of temperature on K_dec_*), decane’s advantage emerges from the thermal acceleration of chain mobilization, enhanced by its lower viscosity and linear structure. Consequently, the *E_a_* values provide a quantitative bridge between molecular-scale solvent characteristics and the macroscopic reversal (temperature-dependent shift) in solvent effectiveness.

The temperature dependence of *K_dec_* and *r_dis_* for PP in decalin and decane, established from experimental data over the 403–443 K range, enables extrapolation across a wider temperature range. Note, however, that the Arrhenius fit for the decrystallization rate constant (*K_dec_*) is valid only below the PP melting point (~166.5 °C (~440 K)). We note that the inclusion of the 170 °C data point in the Arrhenius fit is consistent with the DSC results which indicate that melting is still in progress at this temperature; hence partial crystallinity remains.

### 4.6. Effect of Solvent Type on Decrystallization and Disentanglement

Understanding how the key parameters governing the dissolution of semicrystalline polymers, *K_dec_* and *r_dis_*, depend on solvent type enables more rational solvent selection, grounded in kinetic understanding rather than solubility parameters alone.

Smaller solvent molecules generally exhibit higher diffusivity within the polymer matrix, facilitating faster chain disentanglement and a more effective disruption of crystalline domains [78,79]. Beyond molecular size, solvent shape, such as cyclic versus linear or aromatic versus alicyclic, also plays a crucial role in governing dissolution behavior by influencing both kinetic and thermodynamic factors [25,27]. Our model accounts for all relevant solvent-specific factors. The solvent diffusion coefficient is derived within the model and reflects trends consistent with molecular size (e.g., higher diffusivity for decalin compared to decane). Additional factors, such as molecular shape, are implicitly captured through the fitted parameters *r_dis_* and *K_dec_*.

**Figure 7:**
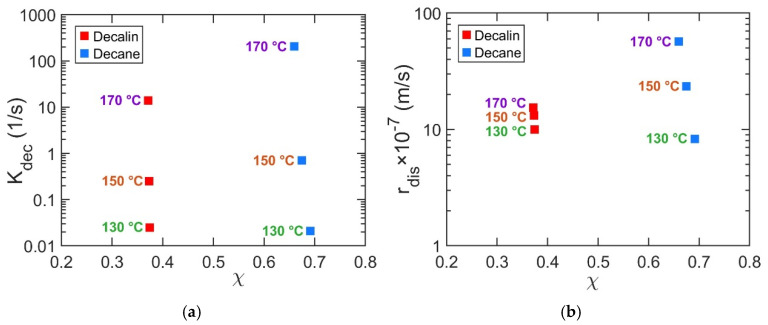
(**a**) *K_dec_* and (**b**) *r_dis_* values plotted versus the polymer–solvent interaction parameter *χ* at 130, 150, and 170 °C.

Among the two solvents studied here, decalin is a bicyclic alicyclic compound and decane is a linear alkane, making direct comparisons nontrivial. According to solubility models, the Flory–Huggins interaction parameter (*χ*) represents polymer–solvent affinity, with lower *χ* values indicating stronger interactions. At 130 °C, the fitted *K_dec_* and *r_dis_* parameters reflect this expectation: decalin, with its lower *χ* and smaller molecular structure, yields higher *K_dec_* and *r_dis_* values than decane (Figure 7). As temperature increases to 150 and 170 °C, however, this trend is inverted, with higher *K_dec_* and *r_dis_* values in decane than in decalin (Figure 7). This inversion suggests a shift in the mechanism governing solvent effectiveness. At lower temperatures, dissolution is favored by smaller, more mobile decalin molecules. At elevated temperatures, increased thermal energy overcomes diffusional constraints and renders the process interaction-limited; here, decane’s linear architecture provides a geometric match to the PP backbone, enabling a more effective solvation of polymer chains. In contrast, the compact, bicyclic structure of decalin is less efficient at this intimate chain–solvent coupling, despite its more favorable *χ* parameter. In addition, solvent–solvent affinity may play a role, as the structural similarity between decane and PP favors solvation, while decalin’s dissimilar geometry may promote self-association (interact with one another) over polymer solvation.

Overall, at moderate temperatures, solvent molecular size appears to play a slightly greater role in influencing *K_dec_* and *r_dis_*, whereas at elevated temperatures, shape becomes increasingly dominant.

### 4.7. Effect of Particle Size on Decrystallization and Dissolution Kinetics

Particle size is a critical operational parameter in the polymer dissolution step in processing and recycling. The systematic variation in PP particle radius that is presented here allows us to study the dominant rate-limiting mechanism. The resulting insights provide a scientific framework for optimizing both feedstock preparation and reactor operation [17,80]. For example, it can enable engineers to balance the energy cost of grinding with the dissolution kinetics of various particle radii, moving from empirical decision-making to predictive, molecular mechanism-based design.

**Figure 8:**
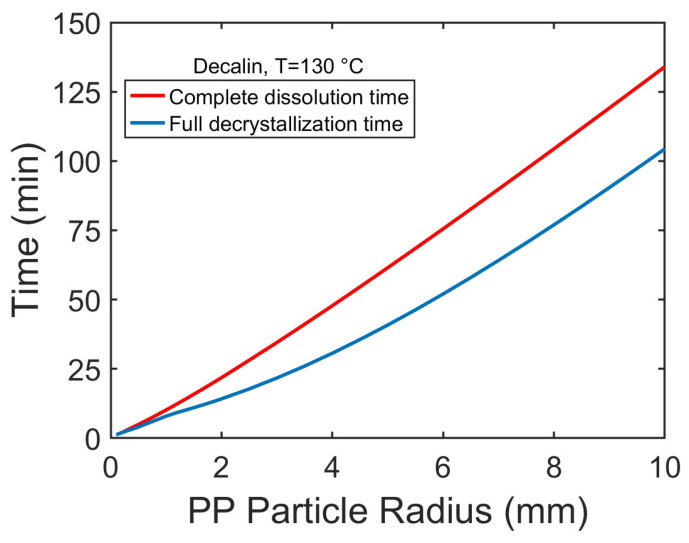
Effect of particle radius on time for full decrystallization and time for complete dissolution of spherical PP pellets in decalin at 130 °C.

Increasing particle radius increases the time required for the full decrystallization and complete dissolution of PP particles (Figure 8), primarily due to longer solvent diffusion pathways. Decrystallization consistently precedes dissolution, but the time gap between the two processes widens with increasing particle size. This suggests that for smaller particles, decrystallization and dissolution occur concurrently, while for larger particles, the dissolution of amorphous polymers, governed by chain disentanglement, becomes the dominant rate-limiting step.

**Figure 9:**
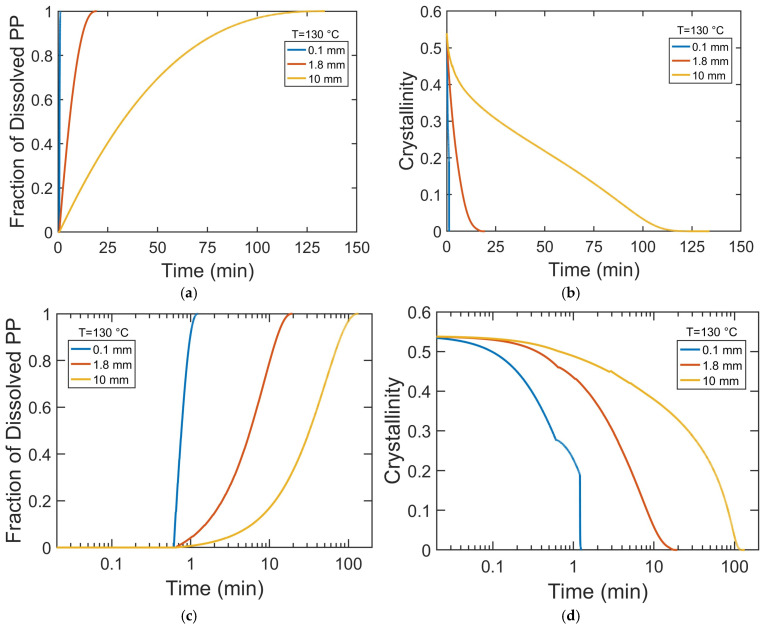

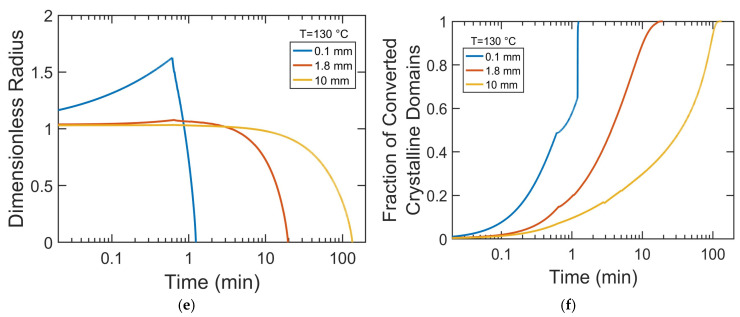
The dissolution kinetics of PP pellets with radii of 0.1 mm, 1.8 mm, and 10 mm in decalin at 130 °C. Plots (**a**,**b**) show the fraction of dissolved PP and the crystallinity of PP during the dissolution process. Plots (**c**,**d**) present the same data on a logarithmic timescale. Plots (**e**,**f**) show the time evolution of dimensionless pellet radius and the fraction of converted crystalline domains.

To gain mechanistic insight into how particle size influences dissolution kinetics, we investigated the time evolution of the swelling, decrystallization, and dissolved fraction of PP pellets with varying radii. This analysis provides direct evidence of whether dissolution is primarily governed by solvent diffusion, crystalline disruption, or chain disentanglement and highlights how the dominant mechanism shifts with particle size. To better visualize early-stage kinetics, the time axis is presented on a logarithmic scale in Figure 9c–f. Figure 9a,c show that the dissolution rate decreases with increasing particle size. In 0.1 mm particles, solvent rapidly saturates their small volume, increasing chain mobility and disrupting crystalline order, resulting in rapid decrystallization (Figure 9b,d,f) and fast dissolution (Figure 9a,c). In contrast, for larger particles (1.8 mm and 10 mm), slower solvent diffusion creates significant concentration gradients, leading to a more gradual decrease in PP crystallinity. The degree of swelling is inversely related to particle radius (Figure 9e). While 0.1 mm pellets swell extensively and dissolve quickly, larger pellets exhibit little swelling and shrink slowly due to longer solvent diffusion paths and a lower surface-area-to-volume ratio. These factors maintain a solvent-poor crystalline core and delay the complete dissolution of the polymer particles.

**Figure 10:**
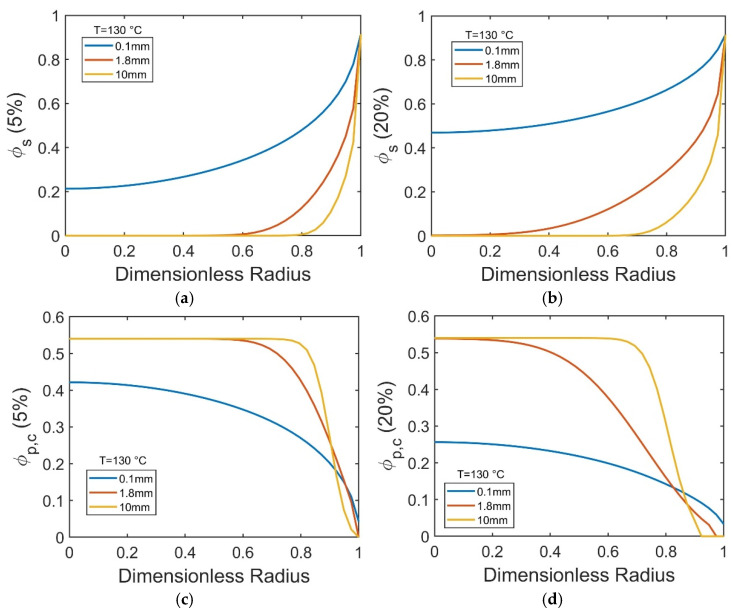
The volume fractions of solvent (∅*_s_*) (top panels) and crystalline domains (∅*_p,c_*) (bottom panels) across the dimensionless radial position of spherical PP pellets with radii of 0.1 mm, 1.8 mm, and 10 mm, shown at 5% (**a**,**c**) and 20% (**b**,**d**) progression toward complete dissolution in decalin at 130 °C.

While Figure 9 highlights the pronounced size dependence of dissolution timescales, the evolving internal structure of the dissolving particles is captured in Figure 10, which reports the spatial distributions of solvent (∅*_s_*) and crystalline polymer (∅*_p,c_*) volume fractions at the early (5%) and intermediate (20%) stages of PP dissolution in decalin at 130 °C.

In small particles (0.1 mm), even at the early stage of dissolution (5%), a high solvent concentration (∅*_s_* ≈ 0.2) is already present in the particle core (Figure 10a), leading to decreased crystallinity across the particle radius (Figure 10c). By the time of 20% progression toward dissolution, both solvent diffusion and polymer decrystallization have advanced further, with ∅*_s_* increasing to ~0.5–0.9 and ∅*_p,c_* dropping below 0.25 throughout the particle (Figure 10b,d). In contrast, for larger pellets (1.8 mm and 10 mm), solvent and corresponding crystallite disruptions take place close to the particle surface, leaving the particle interior solvent-poor and mostly crystalline, at both the 5% and 20% stages (Figure 10a–d). This persistent crystalline core in larger pellets actively resists bulk swelling (Figure 9e) and prolongs the overall dissolution time (Figure 9a,c).

## 5. Conclusions

Polypropylene is produced in large amounts (79 million tons worldwide in 2023, valued at USD 121 billion) [81], and it is used in a variety of applications, including packaging (food containers, films), industrial products (tanks, pallets), automotive parts (e.g., bumpers), consumer goods (toys, appliances), medical devices (e.g., syringes), and textiles (e.g., sportswear). Many PP products are discarded following a single use; however, the recycling rate of PP is in the single digits, leaving a lot of value in landfills. As brands and customers push for the incorporation of recycled content in plastic products, the demand for recycled PP is expected to increase. This provides impetus for companies like PureCycle to produce recycled PP via an advanced recycling process that involves the dissolution of PP waste feedstock, separation of PP in the dissolved state from other compounds, and precipitation of PP from its solution to recover solid PP.

This interesting application of PP dissolution brings in focus the fundamentals of PP dissolution. Following four studies that appeared between 1976 and 1978 [22,23,24,25], a recent study by Ferchichi et al. [26] tracked polypropylene dissolved mass over time using in situ spectroscopic methods but did not address decrystallization during dissolution. This omission is significant in the dissolution of semicrystalline PP: crystalline domains restrict solvent diffusion and polymer chain mobility, so their disruption largely determines when and how dissolution proceeds. Further, their study lacked an analysis of rate-controlling steps, an evaluation of how solvent characteristics and temperature influence these steps, and any connection of empirical observations to a predictive framework.

Given our interest in polyolefin dissolution in the context of dissolution–precipitation recycling [15], and following ongoing joint experimental and modeling work on the dissolution of high-density polyethylene (HDPE) [82], we address here the fundamentals of polypropylene dissolution. Specifically, we present a model that describes the different phenomena involved in the dissolution of semicrystalline PP, validate the model with the experimental results on the decrystallization and dissolution kinetics of PP pellets across different solvents and temperatures, obtain insights into the interplay of decrystallization and polymer chain disentanglement during the time-course of dissolution, and predict particle size effects on dissolution utilizing the validated model.

According to the fit of the model to experimental data on the decrystallization time and dissolved amount kinetics of PP pellets, the dissolution of semicrystalline PP involves the diffusion of the solvent in the solid material, solvent-induced decrystallization, the solvent swelling of amorphous polymer domains, the disentanglement of polymer chains from the solid material, and subsequent diffusion into the solution. Along the way, the PP particle initially swells in size and then shrinks until it completely dissolves. The model provides detailed time-resolved and position-resolved information on composition (i.e., crystalline PP, amorphous PP, and solvent) and solvent diffusivity (which depends on composition) across the dissolving polymer particle, in different solvents and temperatures. Such information is unavailable or difficult to obtain experimentally.

Key parameters that capture decrystallization and polymer chain disentanglement are quantified from the model fit to experimental data. Both the decrystallization rate constant (*K_dec_*) and polymer chain disentanglement rate (*r_dis_*) decrease with increasing temperature following an Arrhenius relationship, with activation energies higher than that for crystallization and comparable to that for melt viscosity, which provide insights into the molecular interactions involved. *K_dec_* and *r_dis_* also decrease with increasing solvent quality, but the solvent effect is weaker than the temperature effect. The knowledge on *K_dec_* and *r_dis_* values and their temperature and solvent dependence that is advanced in this work allows for the prediction of PP dissolution behavior over a wide range of temperatures and solvents, thus facilitating process design in dissolution–precipitation recycling.

Particle size increases both decrystallization and dissolution times, approximately linearly. Decrystallization consistently precedes dissolution, and the time gap between the two processes widens with increasing particle size. This suggests that for smaller particles, decrystallization and dissolution occur nearly simultaneously, while for larger particles, it takes longer for the solvent to diffuse into their interior, leaving the particle interior solvent-poor and crystalline for longer times.

The novelty of this work comprises the validated model that quantitatively describes the physical processes taking place during the dissolution of semicrystalline polypropylene and can predict macroscopic observables (e.g., time for complete dissolution) and reveal microscopic information (e.g., spatial and temporal compositions and solvent mobility within the dissolving particle) for PP dissolution across a range of particle sizes, temperatures, and solvents. This work can be extended to probe the dissolution of PP of varying initial degrees of crystallinity; of PP objects with different geometries, such as films; and of PP in different solvents or mixtures of solvents. The dissolution of semicrystalline polymers other than polypropylene can also be studied. In addition to its fundamental contributions, this work facilitates the design and optimization of a large-scale dissolution–precipitation recycling process that can unlock value from the million tons of PP annually that is currently being landfilled or incinerated following its use.

## Figures and Tables

**Table 3:** A sensitivity analysis of *r_dis_* and *K_dec_* on the predicted times of full dissolution and decrystallization for PP in decalin at 150 °C. Baseline parameter values (*K_dec_* = 0.25 1/s; *r_dis_* = 1.32 × 10^−6^ m/s) correspond to those obtained from fitting the model to experimental data. To assess the influence of each parameter, *K_dec_* and *r_dis_* were varied individually to half and double their baseline values while holding the other parameter constant.

Parameter	Parameter Value	Time of Full Dissolution (min)	Time of Full Decrystallization (min)
*K_dec_* (with *r_dis_* = 1.32 × 10^−6^ m/s)	Baseline: 0.25 1/s	14.0	4.0
	Half: 0.125 1/s	14.1	5.4
	Double: 0.5 1/s	13.9	3.1
*r_dis_* (with *K_dec_* = 0.25 1/s)	Baseline: 1.32 × 10^−6^ m/s	14.0	4.0
	Half: 0.66 × 10^−6^ m/s	25.0	3.8
	Double: 2.64 × 10^−6^ m/s	8.0	4.3

## Data Availability

The original contributions presented in this study are included in the article. Further inquiries can be directed to the corresponding author.
